# Correlation between Oral Hygiene and IL-6 in Children

**DOI:** 10.3390/dj8030091

**Published:** 2020-08-11

**Authors:** Roberto Lo Giudice, Angela Militi, Fabiana Nicita, Giancarlo Bruno, Cristina Tamà, Fabrizio Lo Giudice, Francesco Puleio, Fabrizio Calapai, Carmen Mannucci

**Affiliations:** 1 Department of Clinical and Experimental Medicine, University of Messina, 98100 Messina, Italy; 2 Department of Biomedical and Dental Sciences and Morphofunctional Imaging, University of Messina, 98100 Messina, Italy; amiliti@unime.it (A.M.); fabnicita@unime.it (F.N.); giancarlobruno94@gmail.com (G.B.); cristinatama6@gmail.com (C.T.); fabrizio.logiudice@hotmail.it (F.L.G.); francesco.puleio@live.it (F.P.); f.calapai@gmail.com (F.C.); cmannucci@unime.it (C.M.)

**Keywords:** Interleukin, oral hygiene plaque index, gingival index, tooth brushing

## Abstract

The aim of this study was to evaluate the correlation between marginal gingivitis, oral hygiene parameters, and interleukin-6 (IL-6) levels in gingival crevicular fluid of 40 children. The marginal periodontal pathology was evaluated by gingival index (GI). The status of oral hygiene was estimated by using patient hygiene performance (PHP), brushing frequency (BF), and plaque index (PI). IL-6 levels in gingival crevicular fluid were measured to evaluate the inflammation in marginal gingiva. PHP score showed a significant correlation with GI, BF, and PI. The groups based on PHP ranges were significantly related to IL-6 concentration in crevicular fluid.

## 1. Introduction

Cytokines are small proteins with autocrine, paracrine, and endocrine signaling functions [1]. Interleukin-6 (IL-6) is a cytokine with pro- and anti-inflammatory effects, involved in inflammation response but also in regenerative processes [2]. Human IL-6 is a helical-shaped protein made up of 212 amino acids, and it belongs to the IL-6 cytokines family [3]. IL-6 is produced by T-cells and macrophage cells that induce immune response [4].

The variation in IL-6 levels in different oral diseases has already been evaluated; inflammatory diseases such as periodontitis and oral lichen planus can induce an increase in cytokines levels [5]. Tooth decay, especially in active state, can induce a significant increase in crevicular fluid IL-6 levels in children [6].

Plaque-related gingivitis is an inflammatory disease involving the gingival tissue caused by bacteria [7]. Gingivitis is a reversible pathology highly frequent in children and adolescents [8], although it can affect people at any age [9,10]. The accumulation of dental plaque is a risk factor for the development of gingivitis and periodontal diseases [11].

The most effective approach to gingivitis and periodontal diseases is prevention by applying oral hygiene procedures correctly and by making periodic controls of the periodontal health status.

Oral fluid biomarkers could provide a framework of the marginal periodontal inflammatory status.

The aim of this study was to evaluate the IL-6 levels in gingival crevicular fluid of children with mixed dentition and their correlation with patient hygiene performance index (PHP), gingival index (GI), plaque index (PI), and frequency of tooth brushing (BF).

The research hypothesis was that higher oral hygiene indices scores (PHP, PI) and lower BF correspond to an increase of marginal gingival inflammation (GI) and of IL-6 levels in gingival crevicular fluid. The null hypotheses were that the distribution of IL-6 levels was the same in the three groups based on the PHP ranges and that there was no relation between the oral indices studied and the measured IL-6 levels.

## 2. Materials and Methods

The study included 40 patients from 4 to 16 years old. The procedures were conducted in accordance with the guidelines of the Helsinki Declaration. The study protocol was approved by the Ethics Committee of Azienda Ospedalieri Universitaria “G. Martino” Messina protocol n 18/18 23/04/2018 considering not invasive nature of test carried out.

Informed consent was collected from parents or legal tutors prior to examination.

Exclusion criteria were systemic and autoimmune diseases or the antibiotic intake during last three months.

### 2.1. Evaluation of Clinical Indices

To evaluate hygiene performance and capability to follow toothbrush instructions other indices were recorded including:

1. For PHP, a dental plaque coloring dye was applied (Red-Cote, GUM, Saronno, VA, Italy) and after 30 s the buccal surfaces of 1.6 and 2.6, the lingual surfaces of 3.6 and 4.6, and the labial surfaces of 1.1 and 3.1 were analyzed [12]. In order to evaluate the debris, each tooth surface was divided into five zones: mesial, distal, and medial (gingival, incisal, occlusal area), and a score for each area was assigned (0 = absence; 1 = presence). The sum of the detected values was then divided for the number of teeth examined.

The PHP scores are identified in the Table 1.

2. Toothbrushing frequency (BF) was used as a quantitative indicator of toothbrushing, reported by patient as: never (BF = 0), once a day (BF = 1), or more than once a day (BF > 1).

3. In order to estimate the presence of plaque at the gingival teeth margin, the PI was used. According to the recording protocols of this index, each of six gingival teeth surfaces were examined with a probe and a range of scores of 0–3 was assigned. The mean of values registered for each tooth determines the score of the patient [13] (Table 1).

4. GI was recorded using clinical inspection and probing; the score was calculated with the ratio mean score of the teeth/number of teeth examined [14] (Table 1).

After calculating the PHP score for each subject, the patients were divided in three groups depending on the PHP ranges: “good PHP group” (0–1.7. *n* = 14); “fair PHP group” (1.8–3.4. *n* = 13); and “poor PHP group” (3.5–5.0. *n* = 13).

### 2.2. Evaluation of IL-6

Children were recommended to avoid food or drinks and to not brush their teeth for ≥1 h before intraoral inspection. Gingival crevicular fluid samples were collected using sterile filter papers by an expert operator. Filter papers were unwrapped and stored at 37 °C for 30 min in sterile test tubes containing 0.5 mL of phosphate-buffered saline.

IL-6 concentration was measured by using the Human IL-6 Quantikine ELISA Kit (R&D Systems; Minneapolis, MN, USA). The samples were shaken every 5 min, in order to ease their extraction from the filter paper. All analyses were made following the manufacturer’s protocol. Absorbance was measured by a Wallac 1420 Victor2 multi-label counter (Perkin-Elmer Life Sciences, Turku, Finland) at 450 nm (correction wavelength set at 540 nm).

### 2.3. Statistical Analysis

The numerical data were expressed as mean value ± standard deviation (SD) and the categorical variables as frequency and percentages. Variables examined did not present normal distribution as verified by the Kolmogorov–Smirnov test. Consequently, the non-parametric approach was used for the statistical analysis.

Statistical comparisons between different PHP ranges groups were carried out using the chi-square test for categorical variables and the Kruskal–Wallis test with Bonferroni correction for numerical parameters. Spearman’s (Rho) test was used to calculate statistical correlations between different oral hygiene indices.

A *p*-value < 0.05 was considered statistically significant. Statistical analyses were performed using SPSS for Windows, version 25.0 (IBM, New York, NY, USA).

## 3. Results

**Figure 1:**
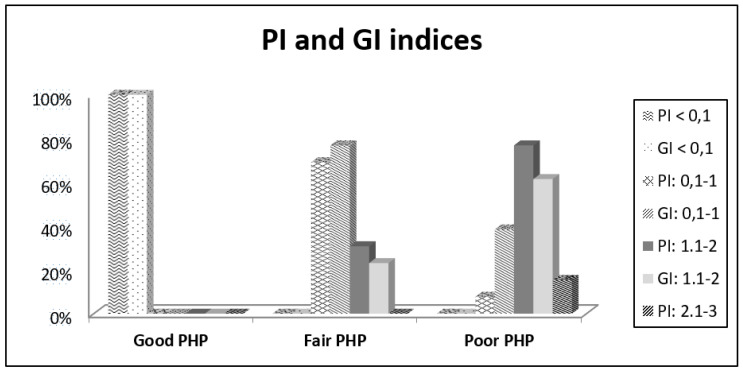
Descriptive statistics (% of gingival index (GI) and plaque index (PI)).

**Figure 2:**
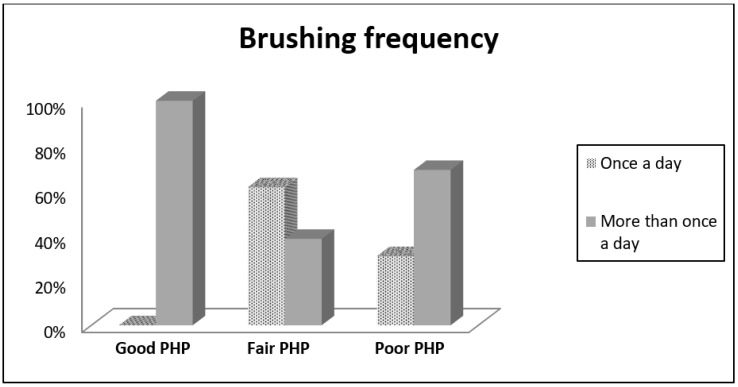
Descriptive statistics (brushing frequency).

The percentage values of the Plaque and Gingival Indices and brushing frequency associated to PHP ranges are reported in Figure 1 and Figure 2.

The mean values and standard deviations for the numerical variables of the PI and GI indices and IL-6 concentrations based on PHP groups are reported in Table 2.

The Kruskal–Wallis test, applied for the numerical variables of PI, GI, and IL-6 concentration, showed that the distribution of the values of the PI and GI indices changed significantly between the PHP groups.

In detail, the difference was statistically significant for the PI and GI values between the subjects who have a good PHP and those who have a fair PHP (*p* = 0.001), and between the subjects who have a good PHP and a poor PHP (*p* < 0.001).

Moreover, this test showed a statistically significant difference regarding the IL-6 concentrations between all groups examined (*p* = 0.008 good PHP Group vs. fair PHP Group; *p* < 0.001 good PHP vs. poor PHP; *p* = 0.014 fair PHP vs. poor PHP).

The brushing frequency was used as a categorical variable and had the following percentages within the PHP groups; 61.5% of the fair PHP group and 30.8% of the poor PHP group carry out oral hygiene once a day, while 100% of good PHP group, 38.5% of fair PHP group, and 69.2% of poor PHP group brush their teeth more than once a day. The chi-square test showed statistically significant differences between the PHP groups (*p* = 0.002).

The correlation between the different oral indices and the brushing frequency was calculated using the correlation index of Spearman (Rho; Table 3).

Spearman’s correlation test showed that PHP index was highly related to PI and GI indices (ρ = 0.958 and ρ = 0.886, respectively). Furthermore, it has a low negative correlation with the brushing frequency (ρ = −0.397). PI index also showed to be highly related to GI (ρ = 0.900) and a low negative correlation to brushing frequency (ρ = −0.453) (Table 3).

Spearman’s test was also evaluated the correlation between oral indices and IL-6 concentration (Table 4).

The PHP and PI indices had a very high positive correlation with IL-6 levels (ρ = 0.940 and ρ = 0.909, respectively). Moreover, IL-6 concentrations were strongly related to GI (ρ = 0.825) and negatively correlated to BF (ρ = −0.412).

## 4. Discussion

The focus of our research was to study the correlation between individual oral hygiene performance quality (PHP) and IL-6 levels in crevicular fluid. The choice of analyzing this type of interleukin is due to the role of cytokines in inflammatory process. IL-6 concentrations in saliva and gingival crevicular fluid could provide information about the gingival inflammatory status.

A poor oral hygiene performance quality in children could lead to plaque accumulation, which induces gingival inflammation and could increase inflammatory biomarkers concentrations in oral fluids. The correlation between oral hygiene, gingival inflammation, and inflammatory response has been largely described [14,15,16,17]. Optimal values of performance hygiene index are significantly related to absence of gingival pathology and caries [6].

In our study, the statistical evaluation of the correlations between PHP and oral hygiene indices (PI and BF) confirms that PHP is significantly related to BF and PI.

In 100% of patients with good PHP, the plaque amount (PI) was significantly lower (<0.1). In cases with lower hygiene performance quality (fair and poor PHP groups), the plaque index significantly increased.

Furthermore, 100% of patients in the good PHP group brushed their teeth more than once a day (BF > 1).

The relation between the brushing frequency and the patient performance index is subverted in the poor PHP group. This data could be related to the low quality of the brushing technique or to a bias that push patients to give the self-convicted more correct answer. This aspect is related to our decision to use objective index instead of reported data.

The acquired data show that PHP is an effective index for the objective expression of oral hygiene performance quality.

Gingival index (GI) is significantly related to PHP. Marginal gingivitis is absent in good PHP Group, and it increases with significant differences in the other groups (fair and poor PHP).

These data confirm that adequate oral hygiene is important to prevent gingival inflammation in children.

Many authors have shown that plaque accumulation is the primary cause of marginal periodontal inflammation [18,19,20,21]. Toothbrushing frequency has also a correlation with Decayed Missed Filled Tooth Index (DMFT), resulting in a lower susceptibility to dental caries development [22,23,24,25,26].

In our research, PHP, PI, and GI was significantly related to IL-6 concentrations in crevicular fluid. BF was also correlated with the amount of IL-6; increasing the frequency of home oral hygiene decreased the concentration of IL-6 in the crevicular fluid.

Ebersole et al. showed that the concentration of salivary IL-6 was much higher in patients with periodontal disease compared to patients with gingivitis and healthy patients [27]. Bartold and Haynes reported higher IL-6 levels in inflamed gingival tissues compared to healthy control tissues [28]. Lo Giudice et al. reported that a high level of IL-6 is present when caries is in an active state and associated to infectious consequences [6].

Furthermore, our statistical data show that the distribution of IL-6 concentrations is significantly different between the three groups (good, fair, and poor PHP) when the Bonferroni correction in the Kruskal–Wallis test was applied; therefore the null hypothesis was rejected.

The data confirm as IL-6 significantly increases in children with poor hygiene performance index, gingival inflammation, and plaque presence.

Thus, the research confirms that gingival inflammation and IL-6 concentrations depend on oral hygiene performance quality and the consequent plaque accumulation. This evidence underlines the importance of plaque control in children.

## 5. Conclusions

Gingival inflammation, plaque accumulation, and a low-quality plaque control can induce an increase of IL-6 levels in crevicular fluid in children.

Our study confirms that IL-6 is associated with oral inflammatory response. This correlation is evident also when studying gingival inflammation and other plaque-related pathologies as reported in previous research. Anyway, further evaluations in crevicular cytokines are necessary to analyze the chemical biomarkers in marginal gingival inflammatory processes, and a bigger sample size could give a stronger statistical relation between the parameters evaluated.

## Figures and Tables

**Table 1:** Oral hygiene parameters. (patient hygiene performance index (PHP), gingival index (GI), plaque index (PI), and frequency of tooth brushing (BF)).

PI	GI	PHP
<0.1: No plaque in the gingival area;	<0.1: No inflammation;	0.0–1.7: Good
0.1–1: Minimal quantity of plaque adhering to the free gingival margin and adjacent area of the tooth.	0.1– 1: Mild inflammation, no bleeding on probing;	1.8–3.4: Fair
1.1–2: Moderate accumulation of plaque within the gingival pocket, on the gingival margin and adjacent tooth surface.	1.1–2: Moderate inflammation, bleeding on probing;	3.5–5.0: Poor
2.1–3: Considerable quantity of plaque within the gingival pocket and gingival margin.	2.1–3: Severe inflammation, tendency to spontaneous bleeding.	

**Table 2:** Descriptive statistics based on PHP groups of PI, GI, and Interleukin-6( IL-6) expressed as mean value with Standard Deviation (SD).

PHP Groups		PI Index	GI Index	IL-6
Good PHP	*n*	14	14	14
	Mean	0.0000	0.0000	3.1821
	SD	0.00000	0.00000	0.85516
Fair PHP	*n*	13	13	13
	Mean	1.0231	0.8462	15.0088
	SD	0.41664	0.28756	4.64050
Poor PHP	*n*	13	13	13
	Mean	1.8000	1.2846	34.1442
	SD	0.75498	0.33627	11.44619
Total	*N*	40	40	40
	Mean	0.9175	0.6925	17.0885
	SD	0.89124	0.59760	14.66324

**Table 3:** Spearman’s ρ test between all oral indices( Toothbrushing frequency (BF), Gingival index (GI, number of patients (Paz)).

	PHP	PI	GI	BF
**PHP**				
Coefficient of correlation	1.000	0.958 **	0.886 **	−0.397 *
Sig. (two-sided CI)		0.000	0.000	0.011
# Paz		40	40	40
**PI**				
Coefficient of correlation	0.958 **	1.000	0.900 **	−0.453 **
Sig. (two-sided CI)	0.000		0.000	0.003
# Paz	40		40	40
**GI**				
Coefficient of correlation	0.886 **	0.900 **	1.000	−0.233
Sig. (two-sided CI)	0.000	0.000		0.148
# Paz	40	40		40
**BF**				
Coefficient of correlation	−0.397 *	−0.453 **	−0.233	1.000
Sig. (two-sided CI)	0.011	0.003	0.148	
# Paz	40	40	40	

** The correlation was significant at the 0.01 level (two-tailed). * The correlation was significant at the 0.05 level (two-tailed).

**Table 4:** Spearman’s ρ test between IL-6 and all oral indices.

	PHP	PI	GI	BF
**IL-6**				
Coefficient of correlation	0.940 **	0.909 **	0.825 **	−0.412 *
Sig. (two-sided CI)	0.000	0.000	0.000	0.008
*N*	40	40	40	40

** The correlation was significant at the 0.01 level (two-tailed). * The correlation was significant at the 0.05 level (two-tailed).
